# The genome sequence of the springtail *Allacma fusca *(Linnaeus, 1758)

**DOI:** 10.12688/wellcomeopenres.19690.1

**Published:** 2023-07-21

**Authors:** Kamil S. Jaron, Matty P. Berg, Jacintha Ellers, Christina N. Hodson, Laura Ross, University of Oxford and Wytham Woods Genome Acquisition Lab, Wellcome Sanger Institute Tree of Life programme, Wellcome Sanger Institute Scientific Operations: DNA Pipelines collective, Tree of Life Core Informatics collective, Darwin Tree of Life Consortium

**Affiliations:** 1 Tree of Life, Wellcome Sanger Institute, Hinxton, England, UK; 2 School of Biological Sciences, Institute of Ecology and Evolution, The University of Edinburgh, Edinburgh, Scotland, UK; 3 Amsterdam Institute for Life and Environment, Section Ecology & Evolution, Vrije Universiteit Amsterdam, Amsterdam, North Holland, The Netherlands; 4 Biodiversity Research Centre, Department of Zoology, The University of British Columbia, Vancouver, British Columbia, Canada

**Keywords:** Allacma fusca, springtail, genome sequence, chromosomal, Symphypleona

## Abstract

We present a genome assembly from an individual male *Allacma fusca *(the springtail; Arthropoda; Collembola; Symphypleona; Sminthuridae). The genome sequence is 392.8 megabases in span. Most of the assembly is scaffolded into 6 chromosomal pseudomolecules, including the X _1_ and X _2_ sex chromosomes. The mitochondrial genome has also been assembled and is 14.94 kilobases in length.

## Species taxonomy

Eukaryota; Metazoa; Eumetazoa; Bilateria; Protostomia; Ecdysozoa; Panarthropoda; Arthropoda; Mandibulata; Pancrustacea; Hexapoda; Collembola; Symphypleona; Sminthuridae; *Allacma*; *Allacma fusca* (Linnaeus, 1758) (NCBI:txid39272).

## Background

**Figure 1.**
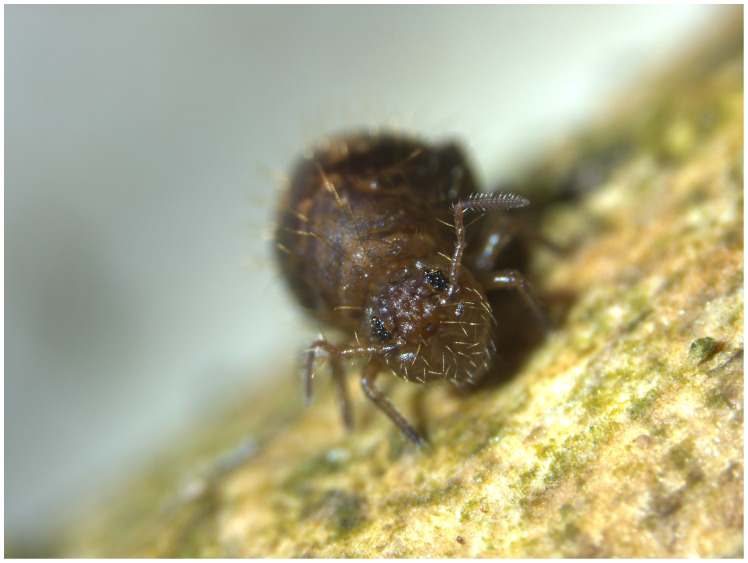
Photograph of an *Allacma fusca* individual from the same locality as the sequenced specimen.

Springtails are one of the most abundant groups of soil animals, found in all sorts of biomes and habitats worldwide ( Hopkin, 1997). *Allacma fusca* is the very first springtail described by De Geer in 1744, which perhaps does not come as a surprise given it is one of the largest springtails in the UK (up to 3.5 mm; Hopkin, 1997). *Allacma fusca* is a brown-coloured ( Figure 1) globular springtail (Symphypleona; Sminthuridae), commonly found on bark of trees overgrown with green algae. It is native to the palearctic region, but more recently found also on the east coast of the Nearctic and Australia ( GBIF, 2023). Tree trunks are a rather unusual habitat for a globular springtail, they usually live in more humid environments closer to the ground - soil and leaf litter. While other springtails breathe through their cuticle, *A. fusca*, adapted for lifestyle on trees, features a thicker cuticle and a complex system of trachea ( Betsch & Vannier, 2009). This is likely an example of convergent evolution to trachea in insects ( Hopkin, 1997).

The genetics of *A. fusca* features several peculiarities. The female karyotype consists of 2 *n* = 12 chromosomes, but the male karyotype is more complex ( Dallai *et al.*, 2000). Just like in females, the male zygote is initially fully diploid (2 *n* = 12), but in very early embryogenesis, two chromosomes often referred to as X _1_ and X _2_ are eliminated. As a result, males show a 2 *n* = 10 karyotype with X _1_X _2_00 sex chromosomes, although they are not the primary mechanism of the sex determination ( Dallai *et al.*, 2000), which is thought to be maternally controlled ( Haig, 1993). The spermatogenesis of males is also unusual: one full haploid set of chromosomes co-segregate into one secondary spermatocyte, while the one other will contain only four autosomes and no X chromosomes ( Dallai *et al.*, 2000). The cell with two missing chromosomes will immediately degenerate, while the cell with the complete haploid set will undergo the rest of meiosis and form two spermatozoa ( Dallai *et al.*, 2000). The sperm contains one parental haplotype only ( Jaron *et al.*, 2022). It is hypothesised that the retained genome is of maternal origin, therefore *A. fusca* more likely reproduces via so-called paternal genome elimination ( Jaron *et al.*, 2022). A similar type of spermatogenesis is found across at least five globular springtail families ( Dallai *et al.*, 1999; Dallai *et al.*, 2000; Dallai *et al.*, 2001; Dallai *et al.*, 2004), and therefore paternal genome elimination is thought to be ancestral to all globular springtails (Symphypleona). The chromosomal genome assembly will contribute to understanding this peculiar reproductive strategy and other unusual features of this species.

## Genome sequence report

The genome was sequenced from one male *Allacma fusca* from a collection at the Ashworth Laboratories, University of Edinburgh. A total of 80-fold coverage in Pacific Biosciences single-molecule HiFi long reads was generated. Primary assembly contigs were scaffolded with chromosome conformation Hi-C data. Manual assembly curation corrected 221 missing joins or mis-joins and removed 23 haplotypic duplications, reducing the assembly length by 1.83% and the scaffold number by 34.93%, and decreasing the scaffold N50 by 18.48%.

**Figure 2.**
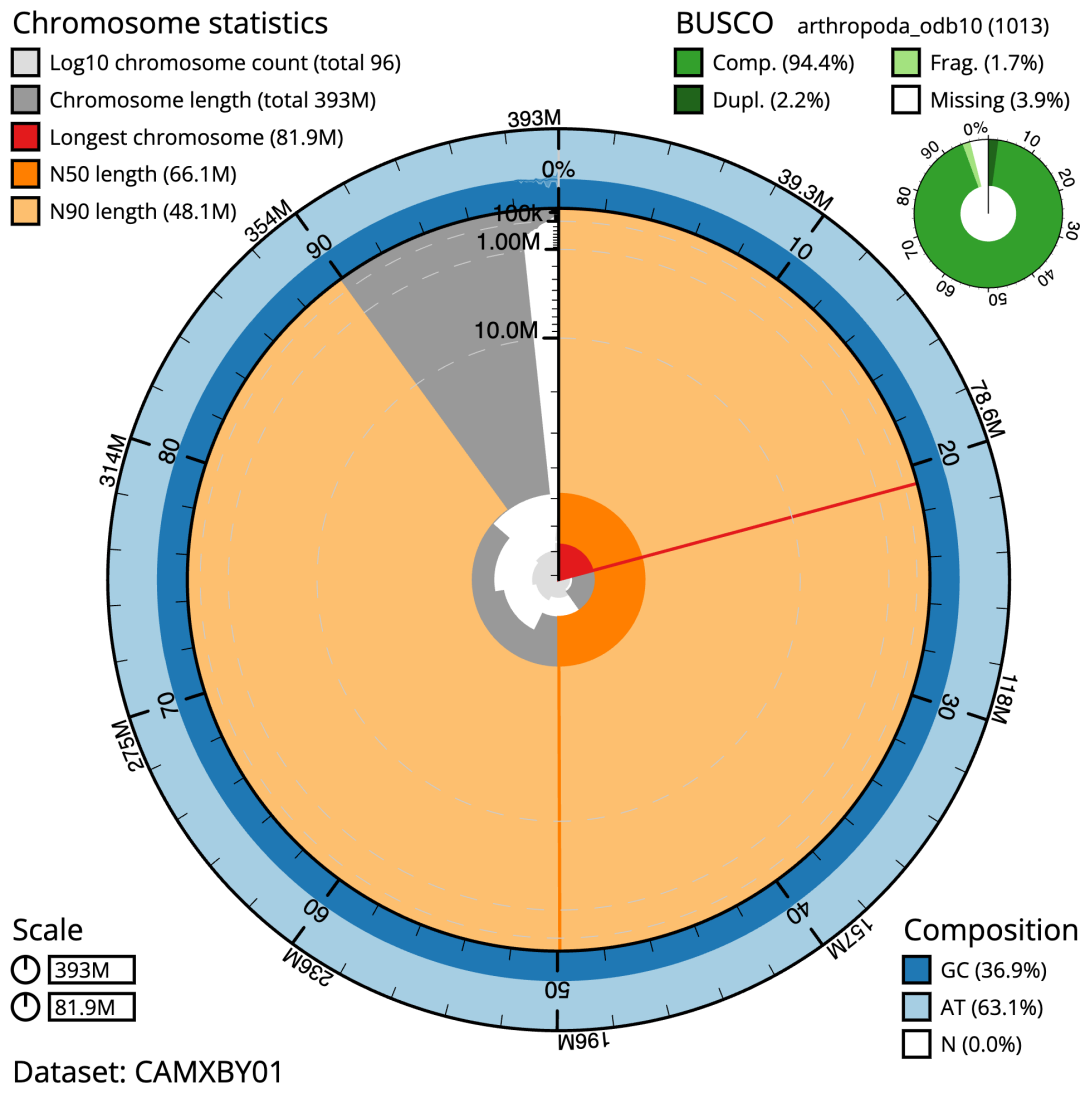
Genome assembly of *Allacma fusca*, qeAllFusc8.1: metrics. The BlobToolKit Snailplot shows N50 metrics and BUSCO gene completeness. The main plot is divided into 1,000 size-ordered bins around the circumference with each bin representing 0.1% of the 392,786,974 bp assembly. The distribution of scaffold lengths is shown in dark grey with the plot radius scaled to the longest scaffold present in the assembly (81,897,997 bp, shown in red). Orange and pale-orange arcs show the N50 and N90 scaffold lengths (66,084,411 and 48,066,046 bp), respectively. The pale grey spiral shows the cumulative scaffold count on a log scale with white scale lines showing successive orders of magnitude. The blue and pale-blue area around the outside of the plot shows the distribution of GC, AT and N percentages in the same bins as the inner plot. A summary of complete, fragmented, duplicated and missing BUSCO genes in the arthropoda_odb10 set is shown in the top right. An interactive version of this figure is available at https://blobtoolkit.genomehubs.org/view/qeAllFusc8.1/dataset/CAMXBY01/snail.

The final assembly has a total length of 392.8 Mb in 95 sequence scaffolds with a scaffold N50 of 66.1 Mb ( Table 1). Most (98.82%) of the assembly sequence was assigned to 6 chromosomal-level scaffolds, representing 4 autosomes and the X _1_ and X _2_ sex chromosomes. A heterozygous inversion was observed on chromosome 3 from ~5,000 kbp to ~45,000 kbp. Chromosome-scale scaffolds confirmed by the Hi-C data are named in order of size ( Figure 2– Figure 5; Table 2). While not fully phased, the assembly deposited is of one haplotype. Contigs corresponding to the second haplotype have also been deposited. The mitochondrial genome was also assembled and can be found as a contig within the multifasta file of the genome submission.

**Table 1.** Genome data for *Allacma fusca*, qeAllFusc8.1.

Project accession data		
Assembly identifier	qeAllFusc8.1	
Species	*Allacma fusca*	
Specimen	qeAllFusc8	
NCBI taxonomy ID	39272	
BioProject	PRJEB53479	
BioSample ID	SAMEA110744450	
Isolate information	qeAllFusc8: PacBio qeAllFusc3: Hi-C	
Assembly metrics *		*Benchmark*
Consensus quality (QV)	58.7	*≥ 50*
*k*-mer completeness	100%	*≥ 95%*
BUSCO **	C:94.4%[S:92.2%,D:2.2%], F:1.7%,M:3.9%,n:1,013	*C ≥ 95%*
Percentage of assembly mapped to chromosomes	98.82%	*≥ 95%*
Sex chromosomes	X _1_ and X _2_	*localised homologous pairs*
Organelles	Mitochondrial genome assembled	*complete single alleles*
Raw data accessions		
PacificBiosciences SEQUEL II	ERR10123885	
Hi-C Illumina	ERR9866431	
Genome assembly		
Assembly accession	GCA_947179485.1	
*Accession of alternate haplotype*	GCA_947179445.1	
Span (Mb)	392.8	
Number of contigs	744	
Contig N50 length (Mb)	1.0	
Number of scaffolds	95	
Scaffold N50 length (Mb)	66.1	
Longest scaffold (Mb)	81.9	

* Assembly metric benchmarks are adapted from column VGP-2020 of “Table 1: Proposed standards and metrics for defining genome assembly quality” from ( Rhie *et al.*, 2021). ** BUSCO scores based on the arthropoda_odb10 BUSCO set using v5.3.2. C = complete [S = single copy, D = duplicated], F = fragmented, M = missing, n = number of orthologues in comparison. A full set of BUSCO scores is available at https://blobtoolkit.genomehubs.org/view/qeAllFusc8.1/dataset/CAMXBY01/busco.

**Figure 3.**
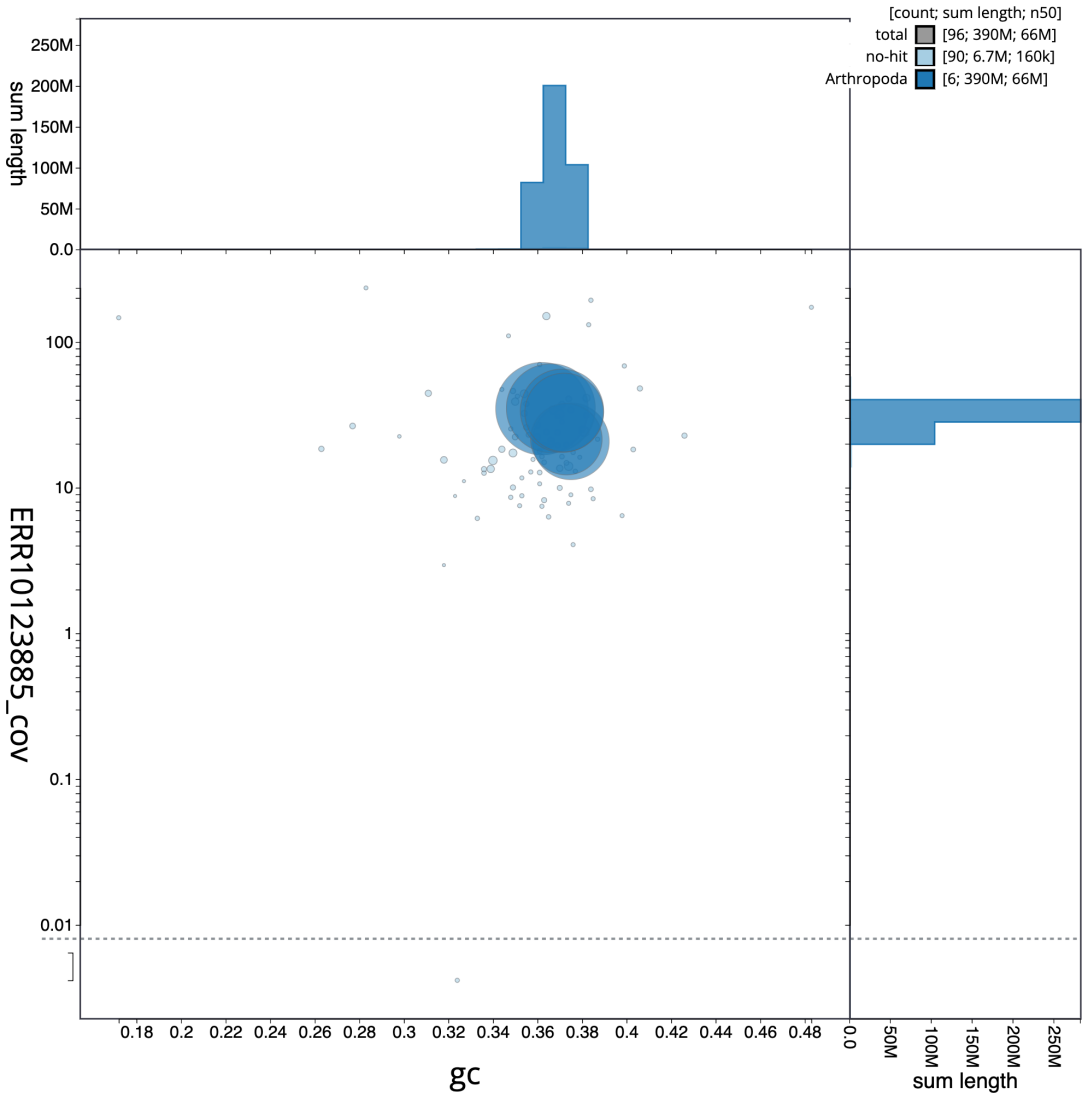
Genome assembly of *Allacma fusca*, qeAllFusc8.1: BlobToolKit GC-coverage plot. Scaffolds are coloured by phylum. Circles are sized in proportion to scaffold length. Histograms show the distribution of scaffold length sum along each axis. An interactive version of this figure is available at https://blobtoolkit.genomehubs.org/view/qeAllFusc8.1/dataset/CAMXBY01/blob.

**Figure 4.**
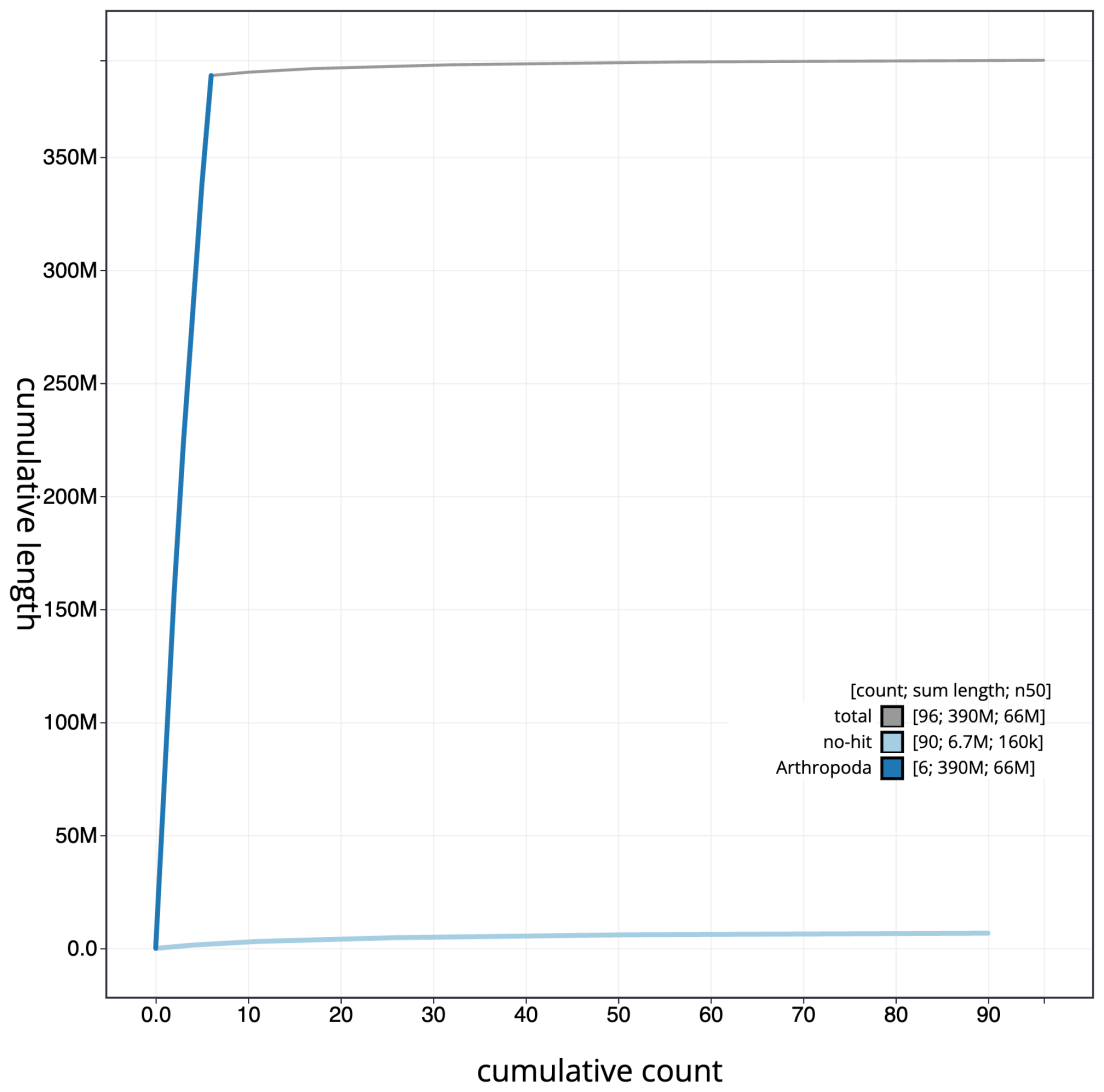
Genome assembly of *Allacma fusca*, qeAllFusc8.1: BlobToolKit cumulative sequence plot. The grey line shows cumulative length for all scaffolds. Coloured lines show cumulative lengths of scaffolds assigned to each phylum using the buscogenes taxrule. An interactive version of this figure is available at https://blobtoolkit.genomehubs.org/view/qeAllFusc8.1/dataset/CAMXBY01/cumulative.

**Figure 5.**
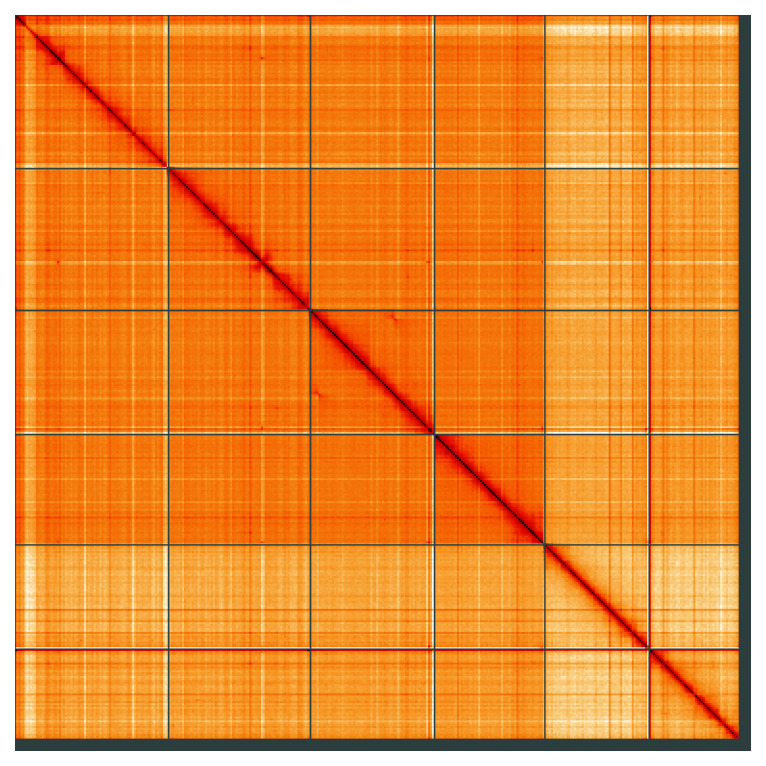
Genome assembly of *Allacma fusca*, qeAllFusc8.1: Hi-C contact map of the qeAllFusc8.1 assembly, visualised using HiGlass. Chromosomes are shown in order of size from left to right and top to bottom. An interactive version of this figure may be viewed at https://genome-note-higlass.tol.sanger.ac.uk/l/?d=ePcubaYsTomWk2nYRGOZXg.

**Table 2.** Chromosomal pseudomolecules in the genome assembly of *Allacma fusca*, qeAllFusc8.

INSDC accession	Chromosome	Length (Mb)	GC%
OX359245.1	1	81.9	36.0
OX359246.1	2	75.61	36.5
OX359247.1	3	66.08	37.0
OX359248.1	4	58.81	37.0
OX359249.1	X1	55.59	37.5
OX359250.1	X2	48.07	37.5
OX359251.1	MT	0.01	28.5

The estimated Quality Value (QV) of the final assembly is 58.7 with *k*-mer completeness of 100%, and the assembly has a BUSCO v5.3.2 completeness of 94.4% (single = 92.2%, duplicated = 2.2%, using the arthropoda_odb10 reference set ( *n* = 1,013).

Metadata for specimens, spectral estimates, sequencing runs, contaminants and pre-curation assembly statistics can be found at https://links.tol.sanger.ac.uk/species/39272.

## Methods

### Sample acquisition and nucleic acid extraction

The specimen selected for this genome assembly was a male *Allacma fusca* (ToLID qeAllFusc8) taken from a collection in the Ashworth Laboratories, University of Edinburgh, which had been aspirated from the bark of various smooth-bark trees. Specimen identification was done using the key ( Hopkin, 2007). The specimens in the collection were kept in a cylindrical plaster cage with mossy bark pieces. The specimen was harvested on 2021-10-04 and frozen from live prior to shipping and sample preparation. 

The specimen used for Hi-C sequencing (specimen number Ox000724, ToLID qeAllFusc3) was collected by Kamil Jaron in Wytham Woods, Oxfordshire (biological vice-county Berkshire), UK (latitude 51.78, longitude –1.34) on 2020-08-02.

DNA for PacBio sequencing was extracted at the Ashworth laboratories, the University of Edinburgh. The qeAllFusc8 sample was frozen in a –70°C freezer. Whole organism tissue was fitted with a BioMasher pestle. High molecular weight (HMW) DNA was extracted using the Salting Out extraction protocol (Steps 2 and 14 of ( christopher.laumer, 2023)). HMW DNA was sheared into an average fragment size of 12–20 kb in a Megaruptor 3 system with speed setting 30. Sheared DNA was size selected using bluePippin. The concentration of the sheared and purified DNA was assessed using a Nanodrop spectrophotometer and Qubit Fluorometer and Qubit dsDNA High Sensitivity Assay kit. Fragment size distribution was evaluated by running the sample on the FemtoPulse system.

### Sequencing

Pacific Biosciences HiFi circular consensus DNA sequencing libraries were constructed according to the manufacturers’ instructions using the ultra-low input protocol. DNA sequencing was performed by Edinburgh Genomics on the Pacific Biosciences SEQUEL II (HiFi) instrument. Hi-C data were also generated from whole organism tissue of qeAllFusc3 using the Arima2 kit and sequenced on the Illumina NovaSeq 6000 instrument at the Sanger Institute.

### Genome assembly, curation and evaluation

Assembly was carried out with Hifiasm ( Cheng *et al.*, 2021) and haplotypic duplication was identified and removed with purge_dups ( Guan *et al.*, 2020). The assembly was scaffolded with Hi-C data ( Rao *et al.*, 2014) using YaHS. The assembly was checked for contamination and corrected as described previously ( Howe *et al.*, 2021). Manual curation was performed using HiGlass ( Kerpedjiev *et al.*, 2018) and Pretext ( Harry, 2022). The mitochondrial genome was assembled using MitoHiFi ( Uliano-Silva *et al.*, 2022), which runs MitoFinder ( Allio *et al.*, 2020) or MITOS ( Bernt *et al.*, 2013) and uses these annotations to select the final mitochondrial contig and to ensure the general quality of the sequence.

A Hi-C map for the final assembly was produced using bwa-mem2 ( Vasimuddin *et al.*, 2019) in the Cooler file format ( Abdennur & Mirny, 2020). To assess the assembly metrics, the *k*-mer completeness and QV consensus quality values were calculated in Merqury ( Rhie *et al.*, 2020). This work was done using Nextflow ( Di Tommaso *et al.*, 2017) DSL2 pipelines “sanger-tol/readmapping” ( Surana *et al.*, 2023a) and “sanger-tol/genomenote” ( Surana *et al.*, 2023b). The genome was analysed within the BlobToolKit environment ( Challis *et al.*, 2020) and BUSCO scores ( Manni *et al.*, 2021; Simão *et al.*, 2015) were calculated.

Table 3 contains a list of relevant software tool versions and sources.

**Table 3.** Software tools: versions and sources.

Software tool	Version	Source
BlobToolKit	4.1.5	https://github.com/blobtoolkit/blobtoolkit
BUSCO	5.3.2	https://gitlab.com/ezlab/busco
Hifiasm	0.16.1-r375	https://github.com/chhylp123/hifiasm
HiGlass	1.11.6	https://github.com/higlass/higlass
Merqury	MerquryFK	https://github.com/thegenemyers/MERQURY.FK
MitoHiFi	2	https://github.com/marcelauliano/MitoHiFi
PretextView	0.2	https://github.com/wtsi-hpag/PretextView
purge_dups	1.2.3	https://github.com/dfguan/purge_dups
sanger-tol/genomenote	v1.0	https://github.com/sanger-tol/genomenote
sanger-tol/readmapping	1.1.0	https://github.com/sanger-tol/readmapping/tree/1.1.0
YaHS	yahs-1.1.91eebc2	https://github.com/c-zhou/yahs

### Wellcome Sanger Institute – Legal and Governance

The materials that have contributed to this genome note have been supplied by a Tree of Life collaborator. The Wellcome Sanger Institute employs a process whereby due diligence is carried out proportionate to the nature of the materials themselves, and the circumstances under which they have been/are to be collected and provided for use. The purpose of this is to address and mitigate any potential legal and/or ethical implications of receipt and use of the materials as part of the research project, and to ensure that in doing so we align with best practice wherever possible. The overarching areas of consideration are:

•   Ethical review of provenance and sourcing of the material

•   Legality of collection, transfer and use (national and international)

Each transfer of samples is undertaken according to a Research Collaboration Agreement or Material Transfer Agreement entered into by the Tree of Life collaborator, Genome Research Limited (operating as the Wellcome Sanger Institute) and in some circumstances other Tree of Life collaborators.

## Data Availability

European Nucleotide Archive: *Allacma fusca*. Accession number PRJEB53479; https://identifiers.org/ena.embl/PRJEB53479. ( Wellcome Sanger Institute, 2022) The genome sequence is released openly for reuse. The *Allacma fusca* genome sequencing initiative is part of the Darwin Tree of Life (DToL) project. All raw sequence data and the assembly have been deposited in INSDC databases. The genome will be annotated using available RNA-Seq data and presented through the Ensembl pipeline at the European Bioinformatics Institute. Raw data and assembly accession identifiers are reported in Table 1.

## Author information

Members of the University of Oxford and Wytham Woods Genome Acquisition Lab are listed here: https://doi.org/10.5281/zenodo.4789928.

Members of the Wellcome Sanger Institute Tree of Life programme are listed here: https://doi.org/10.5281/zenodo.4783585.

Members of Wellcome Sanger Institute Scientific Operations: DNA Pipelines collective are listed here: https://doi.org/10.5281/zenodo.4790455.

Members of the Tree of Life Core Informatics collective are listed here: https://doi.org/10.5281/zenodo.5013541.

Members of the Darwin Tree of Life Consortium are listed here: https://doi.org/10.5281/zenodo.4783558.
