# Supplementary material for: The genome sequence of the springtail Allacma fusca (Linnaeus, 1758)
Source: Wellcome Open Res. 2023 Jul 21;8:319. [Version 1] doi: 10.12688/wellcomeopenres.19690.1 (PMC10427803; doi:10.12688/wellcomeopenres.19690.1)
Supplement: Peer review history

# Peer review: Reviewer response for version 1

**DOI:** 10.21956/wellcomeopenres.21811.r64586

**Published:** 2023-08-15

**Authors:** Francesco Cicconardi

**Affiliations:** 1 School of Biological Sciences, University of Bristol, Bristol, England, UK

In this article, the authors report on the chromosome-level genome assembly of the springtail *Allacma fusca* (Collembola: Symphypleona), one of the few representatives and under-appreciated group of terrestrial arthropods. I’m also glad they picked a male, so they were able to identify the “sex-chromosomes”. This maybe will help to understand the sex determination is these organisms, and if they differ for other non-Symphypleona springtails. The assembly appears to be of good quality using appropriate methods (HiFi long reads, 80-fold coverage, Hi-C data to confirm scaffolding, manual curation of the final assembly) and following the high standard of the Darwin Tree of Life Project. I have only minor comments.

 I don’t think that the sentence “Tree trunks are a rather unusual habitat for a globular springtail, they usually live in more humid environments closer to the ground - soil and leaf litter.” in the background section, is very accurate. In fact, the temperate forests where these springtails live still need to be humid for them to actually be found. Also, there are multiple instances where Symphypleona do not live in a very humid environment, such as the epiphyte *Sminthurus viridis*. I’d change or remove the sentence.

 For the annotation of the genome, it is a bit disappointing that they didn’t attempt to annotate the genome. Because of the almost absolute lack of well annotated springtail genomes, putting some effort from the Darwin Tree of Life Project consortium would have been very beneficial for the whole community.

 Finally, I was wondering why the method section does not list in more detail how the various tools were run (e.g. options).

Are sufficient details of methods and materials provided to allow replication by others?

Partly

Is the rationale for creating the dataset(s) clearly described?

Partly

Are the datasets clearly presented in a useable and accessible format?

Yes

Are the protocols appropriate and is the work technically sound?

Yes

Reviewer Expertise:

Comparative genomics

I confirm that I have read this submission and believe that I have an appropriate level of expertise to confirm that it is of an acceptable scientific standard.

# Peer review: Reviewer response for version 1

**DOI:** 10.21956/wellcomeopenres.21811.r64589

**Published:** 2023-08-15

**Authors:** Daoyuan Yu

**Affiliations:** 1 Soil Ecology Lab, College of Resources and Environmental Sciences, Nanjing Agricultural University, Nanjing, China

The authors report a genome assembly of *Allacma fusca* from a single specimen. The genome size is 392.8 Mb with most of the assembly scaffolded into 6 chromosomal pseudomolecules, including the X1 and X2 sex chromosomes. The mitochondrial genome has also been assembled and is 14.94 kilobases in length. The paper is well-executed and represents a valuable contribution to collembolan genomics. I wholeheartedly recommend it for indexing, following some minor modifications.

 1. Most (98.82%) of the assembly sequence was assigned to 6 chromosomal-level scaffolds, representing 4 autosomes and the X1 and X2 sex chromosomes. Please explain how to identify the X chromosome (X1 and X2).

 2. For a better evaluation of the sequencing quality, please report the N50 of the original PacBio reads in the section 'Genome sequence report'.

 3. The genome was assembled in multiple steps, e.g., assembly was carried out with Hifiasm, haplotypic duplication was identified and removed with purge_dups, and the assembly was scaffolded with Hi-C data using YaHS. Please report the intermediate results and BUSCO completeness at each step of genome assembly.

 4. In the background, the authors claimed that 'Tree trunks are a rather unusual habitat for a globular springtail, they usually live in more humid environments closer to the ground - soil and leaf litter.’ However, it may not be true. Many symphypleones are typical aboveground dwellers, and appear to be rather tolerant to desiccation. They are usually found on living plants, even canopies of tall trees. Beating branches of trees and shrubs often produces abundant specimens of globular springtails (mostly Katiannidae, Bourletiellidae, and Sminthridae). In fact, only neelipleones and some symphypleones (Arrhopalites, some Sminthurinus, etc.) are specialized to a soil life. Probably they would say 'Tree trunks are an unusual habitat for most springtails'.

 5. Also in the background, 'While other springtails breathe through their cuticle, A. fusca, adapted for lifestyle on trees, features a thicker cuticle and a complex system of trachea', sounds like A. fusca is the only springtail species possessing trachea, however, it is not true. Trachea are shared by many symphypleones and a family of elongated springtail: Actaletidae. Perhaps they would like to say 'while MOST other springtails breathe through their cuticle'.

Are sufficient details of methods and materials provided to allow replication by others?

Yes

Is the rationale for creating the dataset(s) clearly described?

Yes

Are the datasets clearly presented in a useable and accessible format?

Yes

Are the protocols appropriate and is the work technically sound?

Yes

Reviewer Expertise:

Entomology, Ecology, Evolution

I confirm that I have read this submission and believe that I have an appropriate level of expertise to confirm that it is of an acceptable scientific standard, however I have significant reservations, as outlined above.

# Peer review: Reviewer response for version 1

**DOI:** 10.21956/wellcomeopenres.21811.r64584

**Published:** 2023-08-11

**Authors:** Nerivânia Nunes Godeiro

**Affiliations:** 1 Research Center, Shanghai Natural History Museum, Shanghai, Shanghai, China

The genome of an intriguing Collembola species with a tracheal system and a peculiar reproductive method was described in the article. Additionally, because *Allacma* specimens are larger than those of other Symphypleona, characterizing its entire genome may be helpful for future research on gene expression. I enjoyed how the results were presented clearly and in interactive charts.

Are sufficient details of methods and materials provided to allow replication by others?

Yes

Is the rationale for creating the dataset(s) clearly described?

Yes

Are the datasets clearly presented in a useable and accessible format?

Yes

Are the protocols appropriate and is the work technically sound?

Yes

Reviewer Expertise:

Phylogenomics of Collembola

I confirm that I have read this submission and believe that I have an appropriate level of expertise to confirm that it is of an acceptable scientific standard.
